# Combining genetic crosses and pool targeted DNA‐seq for untangling genomic variations associated with resistance to multiple insecticides in the mosquito *Aedes aegypti*

**DOI:** 10.1111/eva.12867

**Published:** 2019-09-27

**Authors:** Julien Cattel, Frédéric Faucon, Bastien Le Péron, Stéphanie Sherpa, Marie Monchal, Lucie Grillet, Thierry Gaude, Frederic Laporte, Isabelle Dusfour, Stéphane Reynaud, Jean‐Philippe David

**Affiliations:** ^1^ Laboratoire d’Ecologie Alpine (LECA) UMR 5553 CNRS – Université Grenoble‐Alpes Grenoble France; ^2^ Institut Pasteur de la Guyane Cayenne France

**Keywords:** *Aedes aegypti*, complex phenotype, copy number variations, cytochromes P450s, detoxification enzymes, insecticide resistance, mosquito, polymorphism

## Abstract

In addition to combating vector‐borne diseases, studying the adaptation of mosquitoes to insecticides provides a remarkable example of evolution‐in‐action driving the selection of complex phenotypes. Actually, most resistant mosquito populations show multi‐resistance phenotypes as a consequence of the variety of insecticides employed and of the complexity of selected resistance mechanisms. Such complexity makes the identification of alleles conferring resistance to specific insecticides challenging and prevents the development of molecular assays to track them in the field. Here we showed that combining simple genetic crosses with pool targeted DNA‐seq can enhance the specificity of resistance allele's detection while maintaining experimental work and sequencing effort at reasonable levels. A multi‐resistant population of the mosquito *Aedes aegypti* was exposed to three distinct insecticides (deltamethrin, bendiocarb and fenitrothion), and survivors to each insecticide were crossed with a susceptible strain to generate three distinct lines. F2 individuals from each line were then segregated based on their survival to two insecticide doses. Hundreds of genes covering all detoxifying enzymes and insecticide targets together with more than 7,000 intergenic regions equally spread over mosquito genome were sequenced from pools of F0 and F2 individuals unexposed or surviving insecticide. Differential coverage analysis identified 39 detoxification enzymes showing an increased gene copy number in association with resistance. Combining an allele frequency filtering approach with a Bayesian *F*_ST_‐based genome scan identified multiple genomic regions showing strong selection signatures together with 50 nonsynonymous variations associated with resistance. This study provides a simple and cost‐effective approach to improve the specificity of resistance allele's detection in multi‐resistant populations while reducing false positives frequently arising when comparing populations showing divergent genetic backgrounds. The identification of novel DNA resistance markers opens new opportunities for improving the tracking of insecticide resistance in the field.

## 1 INTRODUCTION

Natural populations experience a variety of selective pressures, leading to the accumulation of locally adaptive features and the expression of complex phenotypes (Orr, 2005). Environmental changes driven by man‐made disturbances can alter the course of selection, by inducing novel, particularly strong and sometimes unpredictable selective pressures. Understanding how natural populations respond to rapid environmental changes has become a major goal, and an increasing number of studies reported adaptive changes on very short timescales (Hendry, Farrugia, & Kinnison, 2008; Hendry, Gotanda, & Svensson, 2017; Palumbi, 2001). Resistance of insects to insecticides is a key example of rapid evolution under novel and strong selective pressures associated with human activities. This adaptive phenotype has evolved quickly and independently in a large number of taxa (Georghiou, 1990). However, natural resistant populations often exhibit complex resistance phenotypes as a consequence of the variety of insecticides used, the variable intensity of selection pressures and the selection of mechanisms conferring resistance to multiple insecticides, making the identification of resistance alleles challenging (Ffrench‐Constant, Daborn, & Le Goff, 2004; Li, Schuler, & Berenbaum, 2007). Besides contributing to the understanding of rapid adaptation and the origins of complex traits, deciphering the complexity of insecticide resistance mechanisms is essential for improving risk assessments and management strategies (Hawkins, Bass, Dixon, & Neve, 2018).

Among taxa of serious economic and medical importance, mosquitoes are vectors of numerous human viruses and pathogens representing a major threat for public health worldwide (Lounibos, 2002). Among them, *Aedes aegypti* (Linnaeus) is of particular importance because of its wide distribution and its capacity to transmit several major arboviral diseases including yellow fever, dengue, Zika fever and chikungunya fever (Brown et al., 2014). Although efforts are invested in developing novel vaccines and strategies to prevent arbovirus transmission, the use of chemical insecticides remains the cornerstone of arboviral diseases control. However, as for malaria vectors, decades of insecticide usage have led to the selection and spread of resistance in this mosquito species. Insecticide resistance is now widespread in *Ae. aegypti* and affects all insecticides used in public health (Moyes et al., 2017), often leading to reduced vector control efficacy (Dusfour et al., 2011; Marcombe et al., 2009, 2011). Although alternative arbovirus control strategies are under development (Achee et al., 2019), their large scale implementation will require decades. Until this, characterizing molecular mechanisms underlying resistance is crucial for tracking down resistance alleles and improving resistance management strategies (Dusfour et al., 2019).

Resistance of mosquitoes to chemical insecticides can be the consequence of various mechanisms, such as nonsynonymous mutations affecting the protein targeted by insecticides, a lower insecticide penetration, its sequestration or its biodegradation often called metabolic resistance (Hemingway, Hawkes, McCarroll, & Ranson, 2004; Li et al., 2007). In *Ae. aegypti*, resistance to pyrethroids, the main insecticide class used against mosquitoes, is mainly the consequence of target‐site mutations affecting the voltage‐gated sodium channel targeted by these insecticides (knock‐down resistance “*kdr”* mutations) and of metabolic mechanisms (Moyes et al., 2017; Smith, Kasai, & Scott, 2016). Several *kdr* mutations have been identified in this species, and the causal association between the V410L, S989P, V1016G/I and F1534C mutations and pyrethroid resistance has been confirmed (Brengues et al., 2003; Haddi et al., 2017; Hirata et al., 2014; Saavedra‐Rodriguez et al., 2007; Yanola et al., 2011). Most of these mutations can be genotyped on individual mosquitoes by PCR‐based assays, providing essential allele frequency data for resistance management. Conversely, metabolic resistance is far less understood in *Ae. aegypti* although this type of resistance usually co‐occurs with target‐site mutations and often accounts for a significant part of the resistance phenotype (Li et al., 2007). Such resistance mechanism is caused by an increased activity of detoxification enzymes, including (but not limited to) cytochrome P450 monooxygenases (P450s or *CYPs* for genes), carboxy/cholinesterases (CCEs), glutathione S‐transferases (GSTs) and UDP‐glycosyltransferases (UDPGTs) (David, Ismail, Chandor‐Proust, & Paine, 2013; Hemingway et al., 2004; Smith et al., 2016). Their high diversity (~300 genes in *Ae. aegypti*) and the complexity of biodegradation pathways make the identification of those conferring resistance to a given insecticide challenging. Theoretically, metabolic resistance can be the consequence of an increased expression of one or multiple detoxification enzymes metabolizing the insecticide and/or the selection of variants showing a higher insecticide metabolism rate due to conformal modifications. Most candidate genes were identified based on their differential transcription in resistant populations as compared to susceptible counterparts using transcriptomics (David et al., 2013; Moyes et al., 2017; Smith et al., 2016; Vontas et al., 2012). Although these approaches identified several detoxification enzymes involved in insecticide biodegradation, they mostly failed to pinpoint their genomic bases, thus impairing the high‐throughput genotyping of metabolic resistance alleles in natural populations. Over the last few years, the application of massive parallel sequencing has improved the understanding of the genetic bases of metabolic resistance in *Ae. aegypti*. By applying deep targeted DNA sequencing on multiple resistant populations from different continents, Faucon et al. (2015) identified several detoxification enzymes affected by copy number variations (CNV) and nonsynonymous variations in association with resistance to the pyrethroid deltamethrin. Cross‐comparing these genomic data with transcriptomic data obtained from RNA‐seq confirmed the central role of CNV in the over‐expression of detoxification enzymes associated with resistance (Faucon et al., 2017). However, as in most studies comparing natural populations, fully discriminating alleles specifically associated with resistance to the insecticide in question from those associated with resistance to other insecticides was not possible. Furthermore, such approach did not allow breaking up genetic linkages between markers, thus potentially leading to false positives arising from hitchhiking effects.

In this context, the present study aims at providing a framework for improving the specificity of resistance allele detection in mosquito populations displaying complex insecticide resistance phenotypes. More precisely, we combined genetic crosses and targeted DNA‐seq in an attempt to identify genomic variations specifically associated with resistance to distinct insecticides in a multi‐resistant *Ae. aegypti* population. After exposure to three insecticides of distinct chemical families (the pyrethroid deltamethrin, the organophosphate fenitrothion and the carbamate bendiocarb), survivors to each insecticide were crossed with a susceptible strain to generate three F2 lines. Individuals from each F2 line were then segregated with two increasing doses of its respective insecticide, and survivors were used to identify CNV and polymorphisms associated with resistance from hundreds of target genes including all detoxification enzymes and insecticide target proteins. In addition, the inclusion of thousands of intergenic regions regularly distributed over mosquito genome in the targeted regions enabled these data to be aligned with a genome‐wide screening of selection signatures associated with resistance. Overall, this study contributes to improve our understanding of the complex genomic bases of resistance to insecticides and provides new opportunities for developing novel DNA‐based insecticide resistance tracking tools in this major arbovirus vector.

## 2 MATERIALS AND METHODS

### 2.1 Mosquitoes

A multi‐resistant composite *Ae. aegypti* population from French Guiana was used in this study, consisting of a pool of six natural populations collected in 2016 in the following localities: Cayenne (North‐East), Sinnamary (North‐East), Saint‐Laurent du Maroni (North), Apatou (North‐West), Maripasoula (West) and Saint‐Georges (East). Each population was collected as larvae from up to five breeding sites located within a 5 km range. These populations were separately raised to the adult stage and blood‐fed to generate adults of the next generation. The composite F0 Guy‐R population was then created by pooling 1,000 virgin adults of both sexes from each population and breeding them together for three generations without insecticide selection.

### 2.2 Controlled crosses

**Figure 1:**
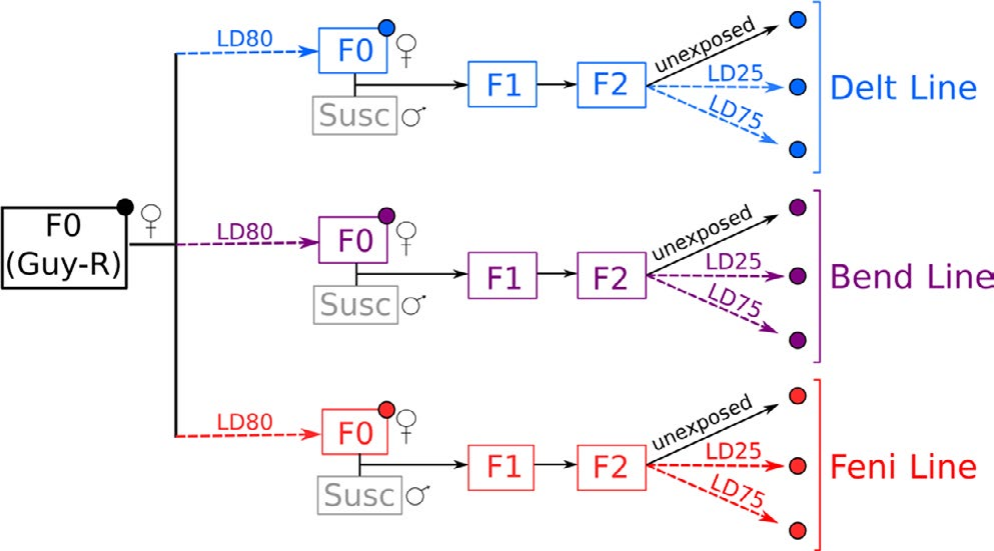
Experimental design overview. Insecticide exposure steps are shown as dashed arrows with the corresponding lethal dose (LD) indicated. Colours indicate insecticide lines (blue: Delt line, purple: Bend line, red: Feni line). The initial resistant composite population (F0 Guy‐R) and the susceptible strain (Susc) are shown in black and grey, respectively. Large dots indicate samples used for targeted DNA‐seq

Virgin F0 Guy‐R females were exposed to a dose killing 80% of individuals (LD_80_) of three insecticides belonging to distinct chemical families: the pyrethroid deltamethrin, the organophosphate fenitrothion and the carbamate bendiocarb. Exposure conditions were identical as for bioassays (see below). Females surviving to each insecticide were then crossed with the fully susceptible strain Bora‐Bora (Susc) in order to create three distinct lines (Figure 1). For each line, controlled crosses were repeated twice and consisted of mass‐crossing 100 virgin females surviving each insecticide exposure (F0‐Delt_LD80_, F0‐Bend_LD80_, F0‐Feni_LD80_) with an equal number of virgin males from the susceptible strain. For each line, F1 individuals were allowed to reproduce freely and blood‐fed in order to generate F2 individuals. F2 individuals from each line were then segregated based on their resistance phenotype by exposing 3‐day‐old females to two increasing doses of their respective insecticide killing 25% and 75% of F2 individuals (LD_25_ and LD_75_). F0 and F2 individuals from each line, unexposed and surviving insecticides, were used for molecular analyses (Figure 1).

### 2.3 Bioassays

All bioassays were performed on 3‐day‐old non‐blood‐fed females using test tubes equipped with insecticide‐impregnated filter papers following WHO guidelines (WHO, 2006). Dose–response bioassays with deltamethrin, fenithrothion and bendiocarb were performed on the F0 Guy‐R composite population to assess its multi‐resistance phenotype and identify the LD_80_ to be used for the selection of most resistant F0 Guy‐R individuals to each insecticide. These bioassays were conducted with at least five doses of deltamethrin (0.05%–1%), fenitrothion (0.0125%–0.4%) and bendioacarb (0.2%–2%) and an exposure time of 60 min. At least five batches of 20 mosquitoes were used for each insecticide dose. Mortality data were recorded after a 24‐hr recovery time and submitted to a probit analysis using the XL‐Stat Excel module (Addinsoft) for estimating LD values. Resistance ratios (RR_50_) to each insecticide were computed from LD_50_ values obtained for each line as compared to the susceptible strain. The LD_80_ used to select most resistant F0 Guy‐R individuals to each insecticide were then defined as follows: deltamethrin 2% for 135 min, bendiocarb 0.5% for 80 min and fenitrothion 1% for 50 min.

Resistance levels of F1 and F2 individuals from each line were obtained following the same procedure. These bioassays allowed identifying the LD_25_ and LD_75_ used to segregate F2 individuals from each line with their respective insecticide as follows: F2‐Delta LD_25_: deltamethrin 0.01% for 60 min; F2‐Delta LD_75_: deltamethrin 0.05% for 60 min; F2‐Bendio LD_25_: bendiocarb 0.2% for 60 min; F2‐Bendio LD_75_: bendiocarb 0.3% for 90 min; F2‐Feni LD_25_: fenitrothion 0.25% for 60 min; and F2‐Feni LD_75_: fenitrothion 0.4% for 60 min.

Cross‐resistance profiles of F2 individuals from each line to all insecticides were evaluated using single‐dose bioassays. For each insecticide, the dose was calibrated in order to obtain a mortality ranging from 20% to 40% in the corresponding F2 line. Doses used were as follows: deltamethrin 0.05% for 60 min, bendiocarb 0.5% for 60 min and fenitrothion 0.1% for 45 min. At least four batches of 20 three‐day‐old non‐blood‐fed females were used per line and insecticide. Mortality was recorded after a 24‐hr recovery time, and data were expressed as mean % mortality ± *SD*. Resistance levels to each insecticide were compared across lines using a generalized linear mixed model (binomial family) using R version 3.5.2 (R Core Development Team).

### 2.4 Deep targeted DNA sequencing

#### 2.4.1 Sample preparation

Deep targeted DNA pool sequencing was used to search for genomic variation associated with insecticide resistance in each line. Genomic DNA was extracted from 2 batches of 50 adult females from each condition (F0 Guy‐R, F0_LD80_, F2_LD25_ and F2_LD75_, see Figure 6) using the PureGene kit (Qiagen) following manufacturer's instructions. DNA extracts obtained from each batch were quality‐checked on agarose gel, quantified using the Qubit dsDNA Broad Range kit (Qiagen) and mixed in equal quantity in order to obtain a single genomic DNA extract representative of 100 individuals for each condition.

#### 2.4.2 Capture of target regions and sequencing

The capture of genomic regions of interest was performed using the SureSelect^®^ target enrichment system (Agilent Technologies). Capture probes were designed based on Aaeg L3 genome assembly and Aaeg L3.3 annotation and consisted in 54,538 overlapping RNA probes of 120 bp. Among them, 32,494 probes targeted the exons and 1.5 kb upstream regions of 336 candidate genes with a mean coverage of 4×. The remaining 22,044 probes targeted 7,348 unique 220 bp intergenic regions equally spread over *Ae. aegypti* genome. Candidate genes were identified from their vectorbase annotation and included all known detoxification enzymes (cytochrome P450s, glutathione S‐transferases, carboxylesterases, UDP‐glycosyltransferases) together with other enzymes potentially involved in insecticide biodegradation pathways and insecticide target proteins. Intergenic regions were defined in order to cover >95% of *Ae. aegypti* genome using the following criteria: target region size = 220 bp; optimal distance between 2 regions = 150 ± 10 kb; region distance to any annotated gene >5 kb; avoid repeated and redundant regions; avoid regions with GC richness >70% or single nucleotide richness >50%; avoid regions with undefined nucleotides (N); do not consider supercontigs <150 kb; and avoid regions located within 75.5 kb of supercontig boundaries. All genomic regions targeted by the study are detailed in Table S1.

Capture was performed with the SureSelect^XT^ Reagent kit (Agilent Technologies) following the “SureSelect^XT^ Target Enrichment System for Illumina Paired‐end Sequencing Library” protocol vB.4. A single genomic DNA extract representative of 100 individuals for each condition was used for capture and sequencing. Briefly, 3 µg of genomic DNA from each sample was fragmented using a Bioruptor (Diagenode), purified, ligated to adaptors and amplified by PCR using Herculase II DNA polymerase (Agilent Technologies). After QC of library size and quantity, libraries were hybridized to biotinylated baits and purified using Dynal MyOne streptavidin beads (Invitrogen). Captured DNA fragments were amplified, purified and multiplexed before sequencing. Sequencing was performed on an Illumina NextSeq500. More than 300 million 75 bp paired reads were generated with an average of 23.3 million reads per sample. Reads were assigned to each sample (unplexing), and adaptors were removed. Reads quality was checked for each sample using FastQC (http://www.bioinformatics.babraham.ac.uk/projects/fastqc), and reads were loaded into Strand NGS v3.1.1 (Strand Life Science) for further analyses.

#### 2.4.3 Reads mapping and filtering

In order to minimize false positives arising from mapping bias in high‐redundancy and low complexity regions, CNV were identified from coding regions. Reads were mapped against all Aaeg L5 exons using the following parameters: padding = 35 bp, minimum identity = 90%, maximum gap = 5%, mean insert size = 167 ± 30 bp, mismatch penalty = 4, gap opening penalty = 6, gap extension penalty = 1, clipping penalty = 5, min align read length = 30 and ignore reads with more than 5 matches, trim 3′ end if base quality <25.

In order to minimize false positives, reads were then filtered to only retain those showing high sequencing quality and mapping quality. The following Strand NGS criteria were used: mean read quality ≥28, N allowed ≤2, alignment score ≥90, mapping quality ≥40 and read length ≥35. Duplicated reads and nonprimary multiply mapped reads were removed as well as inter‐chromosomal split reads, reads with mate missing or mapping to a different chromosome.

For polymorphism analysis, reads were mapped against the whole Aaeg L5 genome in order to consider both genic and intergenic target regions and maximize genome coverage for the detection of selection signatures. The same mapping and filtering parameters as for CNV analysis were applied.

#### 2.4.4 CNV detection

The coverage of all exonic regions was computed, and only regions showing a mean coverage between 30 and 800 reads/bp in all samples and a length >45 bp were retained in order to limit quantification biases. Exon coverages were then normalized according to library size and used for computing normalized copy number values relative to a common reference made from all samples. Normalized copy number values were then averaged per gene and centred‐reduced to minimize stochastic variations. For each insecticide line, genes were considered affected by CNV associated with insecticide resistance if their normalized copy number profile satisfied the following conditions:

$$\left(F0_{\text{LD}80}-F0\right)>0.3\;\text{AND}\;\left(F0_{\text{LD}80}-F2\right)>0\;\text{AND}\;\left[\left(F2_{\text{LD}25}-F2\right)>0.2\;\text{OR(F}2_{\text{LD}75}-F2)>0.2\right].$$

Basically, normalized gene copy number was expected to increase from F0 to F0 survivors, decrease from F0 survivors to F2 after crossing with the susceptible strain and increase in F2 survivors. No dose–response condition was applied to F2 surviving LD_25_ and LD_75_ in order to allow the detection of CNV having a moderate (but potentially additive) effect on phenotype and minimize the confounding effect of target‐site mutations.

#### 2.4.5 Polymorphisms and selection signatures

Variants were called against the whole Aaeg L5 genome using the following parameters: locus coverage >30 in all conditions, confidence calling score cut‐off = 100, ignore loci with homopolymer stretch >4, ignore loci with average base quality ≤15, ignore loci with strand bias ≥50 and coverage ≥50, ignore reads with mapping quality ≤20 and ignore variants with less than 4% supporting reads. Among all variants called, only those polymorphic among our conditions (i.e., showing ≥5% variation between at least one pair of conditions) were retained, and their genic effects were computed.

Associations between polymorphisms and resistance to each insecticide were assessed by combining an allele frequency filtering approach with an *F*_ST_‐based approach.

The frequency filtering approach was based on the expected resistance allele frequency variations across F0 and F2 conditions taking into account their initial frequency. Frequency thresholds used are shown in Table 1. Basically, the frequency of alleles positively associated with resistance was expected to increase from unexposed F0 individuals to F0 survivors, decrease from F0 survivors to unexposed F2 individuals (following crossing with the susceptible strain) and increase again in F2 survivors in association with the insecticide dose. Different initial allele frequency thresholds were used for identifying alleles associated with deltamethrin resistance from those associated with bendiocarb and fenitrothion resistance to account for the higher deltamethrin resistance level of the initial F0 Guy‐R population. The frequency of deleterious alleles (i.e., those negatively associated with resistance) was expected to behave reciprocally.

**Table 1:** Conditions used for identifying polymorphisms associated with resistance

Line^a^	Initial allele frequency (%)^b^	Minimum allele frequency variation^b^				
		F0 to F0_LD80_	F0_LD80_ to F2	F2 to F2_LD25_	F2_L25_ to F2_LD75_	F2 to F2_LD75_
Delt	30–85	+15%	Decrease	Increase	Increase	+15%
	85–90	+10%				
	90–95	+5%				
	>95	Increase				
Bend Feni	15–85	+15%	Decrease	Increase	Increase	+15%
	85–90	+10%				
	90–95	+5%				
	>95	Increase				

a Different initial allele frequency thresholds were chosen for Bend and Feni lines to account for the lower resistance of the initial F0 Guy‐R population to these two insecticides.

b Reciprocal conditions were used for deleterious alleles.

The *F*_ST_‐based approach aimed at assessing departure from neutrality using the Bayesian method implemented in BayeScan version 2.1 (Foll & Gaggiotti, 2008). Because substitutions and deletions may have different probability of occurrence, only substitutions were considered for this analysis. For each insecticide line, two analyses were run separately: the first one contrasting allele frequencies in F0 samples (unexposed and insecticide survivors: F0 Guy‐R and F0_LD80_) and the second one contrasting F2 samples (unexposed and survivors to each insecticide dose: F2, F2_LD25_, F2_LD75_). The Markov chain Monte Carlo (MCMC) algorithm was run with prior odds of 10. The proposal distributions for parameters were adjusted by running 20 short pilot runs of 2,000 iterations. A burn‐in period of 100,000 iterations was used, and the posterior probabilities were estimated from the following 500,000 iterations (10,000 iterations samples every 50). Genomic regions showing low Bayscan *Q*‐values in both F0 and F2 analyses and also including polymorphisms identified by the frequency filtering approach were considered under selection in association with insecticide resistance.

### 2.5 Kdr mutations genotyping

Allelic frequencies for the three kdr mutations (V410L, V1016I and F1534C) initially present in the F0 Guy‐R composite population were inferred for F0 and F2 samples from each line based on reads data. In order to validate allele frequencies obtained from read data, the two kdr mutations V1016I and F1534C were also genotyped in individual mosquitoes from the initial F0 Guy‐R population (F0) and the Delt line. Total genomic DNA was extracted using cetyl trimethyl ammonium bromide chloroform/isoamyl alcohol from 30 non‐blood‐fed females per condition as described in Collins et al. (1987). Individual genotypes for each kdr mutations were obtained by high resolution melt curve qPCR method using 0.15 ng of genomic DNA per reaction as described in Saavedra‐Rodriguez et al. (2007).

## 3 RESULTS

### 3.1 Insecticide resistance levels

**Figure 2:**
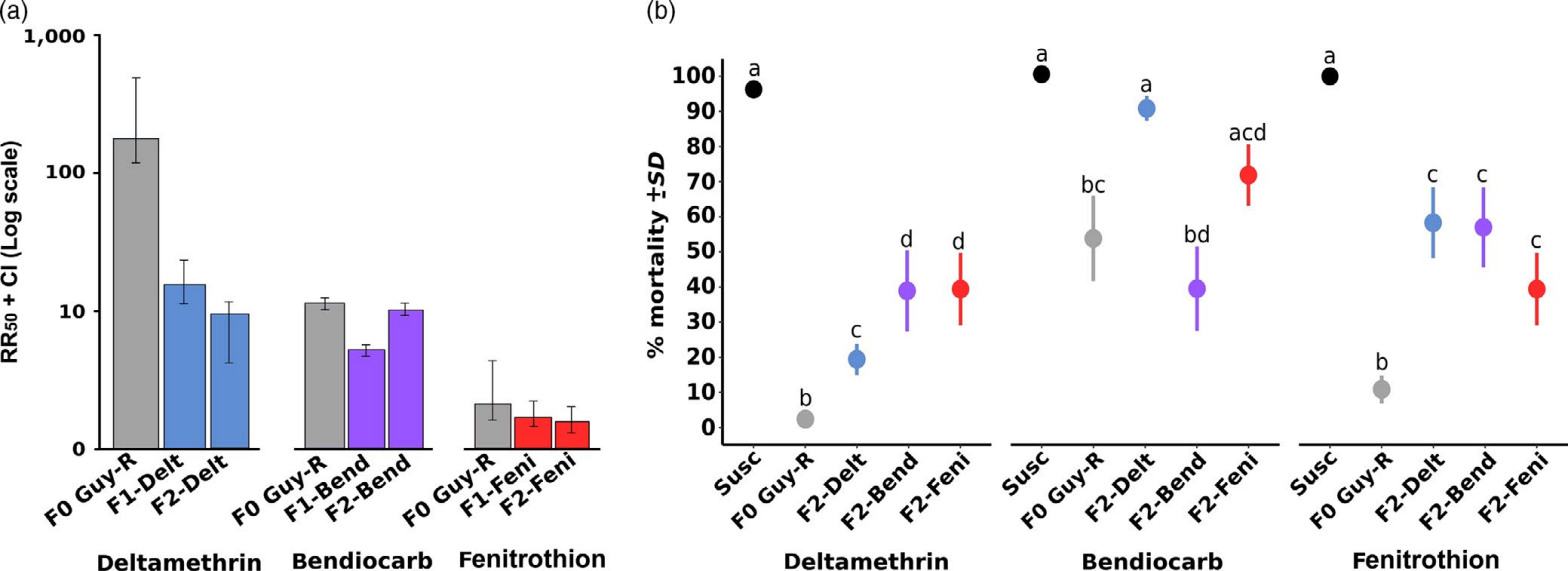
Insecticide resistance levels. Resistance levels of the different lines to the three insecticides deltamethrin, bendiocarb and fenitrothion. Black: susceptible strain, grey: F0 Guy‐R composite population, blue: Delt line, purple: Bend line and red: Feni line. (A) Resistance levels of each line to its respective insecticide at the F0 Guy‐R, F1 and F2 generations. Resistance levels are expressed as LD_50_ ± 95% CI. (B) Cross‐resistance profiles of each line to all insecticides at the F2 generation. Cross‐resistance levels are expressed as % mortality ± *SD* to a single insecticide dose. For each insecticide, letters indicate statistical similarity or dissimilarity between lines (GLM family = binomial, *N* ≥ 4)

Bioassays performed on the initial composite population (F0 Guy‐R) confirmed its high resistance to the pyrethroid insecticide deltamethrin with resistance ratio (RR_50_) over 316‐fold as compared to the susceptible strain (Figure 2A). The F0 Guy‐R population also showed resistance to the carbamate bendiocarb and moderate resistance to the organophosphate fenitrothion with RR_50_ of 14‐fold and threefold, respectively. As expected, resistance to each insecticide decreased after crossing F0 survivors with the susceptible strain, with F1 resistance ratios decreasing to 25‐fold, sevenfold and twofold for deltamethrin, bendiocarb and fenitrothion, respectively. Deltamethrin and fenitrothion resistance moderately decreased in F2 while bendiocarb resistance increased. As expected from controlled crosses, the probit dose–mortality models confirmed the higher heterogeneity of F2 individuals as compared to F1 individuals (Figure S1). Assessing the cross‐resistance level of each line to all insecticides confirmed that each F2 line was enriched in resistance alleles to its respective insecticide (Figure 2B). F2 individuals from each line showed a higher survival when exposed to its respective insecticide although this trend was not significant for bendiocarb and fenitrothion in link with lower resistance levels.

### 3.2 Target‐site resistance mutations

**Figure 3:**
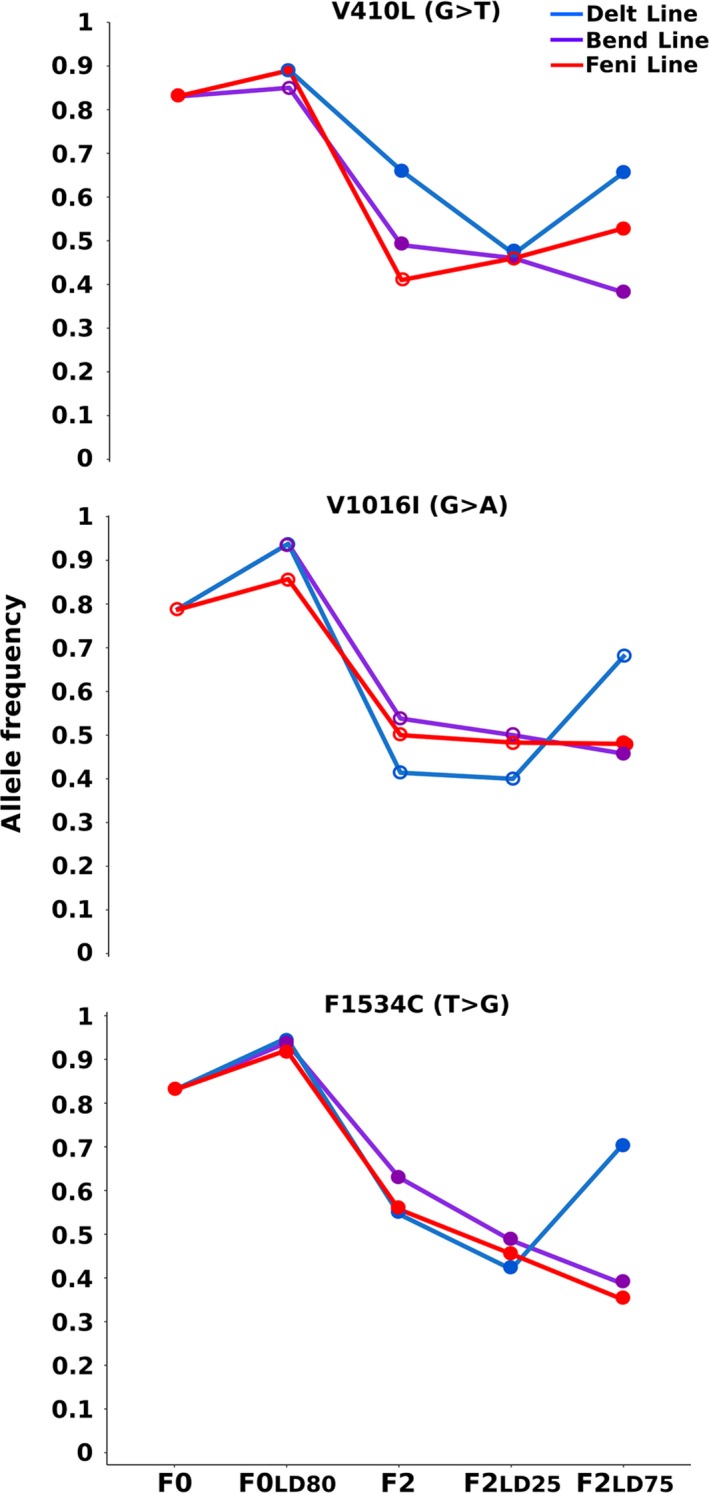
Evolution of kdr mutations frequencies. Allelic frequency variations of the three kdr mutations V410L, V1016I and F1534C initially present in the F0 Guy‐R population in each line. Allele frequencies were inferred from the number of sequencing reads supporting each allele at each locus. Empty dots indicate conditions with read coverage <30

Assessing kdr mutations frequencies from reads data confirmed the high frequency of the three kdr mutations V410L, V1016I and F1534C in F0 Guy‐R composite population, corroborating its high deltamethrin resistance level (Figure 3). Exposing F0 Guy‐R individuals to each insecticide did not lead to differences of Kdr mutation frequencies in survivors. The impact of controlled crosses on the segregation of Kdr mutations became more evident in F2 individuals where higher Kdr frequencies were observed in individuals of the Delt line surviving a high dose of deltamethrin. The acetylcholinesterase G119S mutation conferring resistance to organophosphates and carbamates was not detected in our lines, likely because of the two successive mutation events required in *Ae. aegypti* (Weill et al., 2004). Validation of kdr mutation frequencies on individual mosquitoes by qPCR confirmed the robustness of allele frequencies estimated from reads data although moderate discrepancies were observed when the number of genotyped mosquitoes was low (Figure S2).

### 3.3 Gene copy number variations

**Figure 4:**
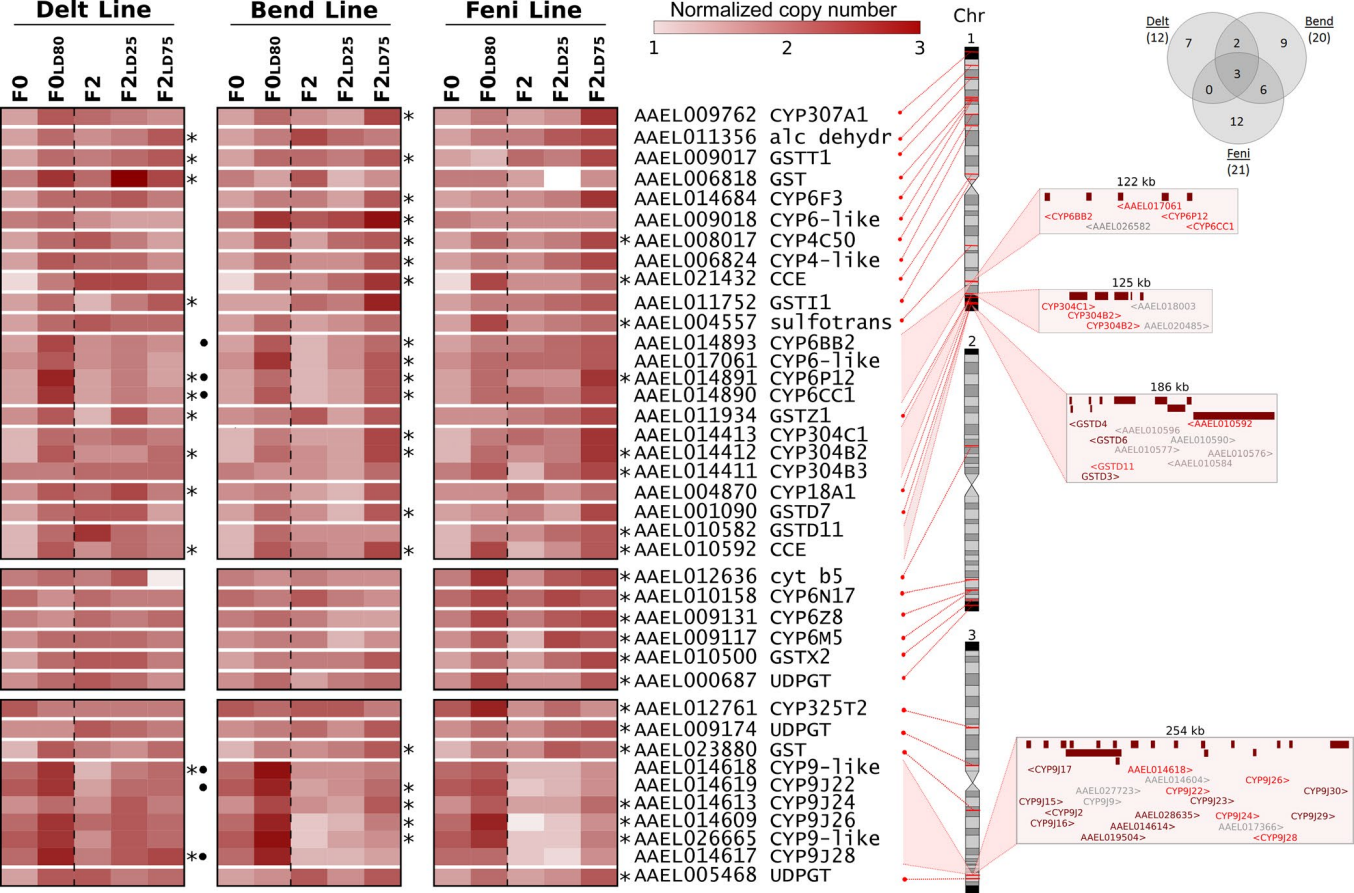
Gene copy number variations associated with insecticide resistance. The heat map shows the normalized copy number profiles of all genes for which an increase CNV was associated with resistance in at least 1 line. For each line, genes affected by CNV associated with resistance are indicated by stars. Data obtained from the initial F0 Guy‐R are shown for each line for better clarity. Black dots indicate genes previously identified as affected by CNV associated with deltamethrin resistance in Faucon et al. (2015). The genomic location of each gene on chromosomes and gene clusters architecture is shown on the right (red: CNV associated with resistance, brown: CNV not associated with resistance, grey: genes not included in the targeted regions). The Venn diagram indicates the number of genes affected by CNV associated with resistance in each line

Over 49.5% of sequenced reads were successfully mapped to the AaegL5 exome allowing the detection of 1,317 exonic regions (719 distinct genes) showing a minimum length of 45 bp and a coverage between 30 and 800 reads/bp in all conditions (median = 94.1 reads/bp). Filtering genes based on their expected CNV profiles across all conditions in each line (increase from F0 to F0 survivors, decrease from F0 survivors to F2 and increase from F2 to F2 survivors, see Section 2 for detailed filtering conditions) allowed identifying 39 detoxification genes affected by CNV in association with insecticide resistance (Figure 4 and Table S2). Although the resistance level of the Delt line was high, more CNV were detected in the two other lines likely due to the contribution of kdr mutations in the deltamethrin resistance phenotype. The confounding effect of kdr mutations also impacted CNV intensities as most genes identified in the Delt line showed a lower CNV increase in F2 individuals surviving high dose of insecticide as compared to those identified in the Bend and Feni lines. Among genes affected by CNV, the P450s *CYP6P12* and *CYP304B2* and the CCE *AAEL010592* were associated with resistance to all insecticides.

Among genes affected by CNV associated with deltamethrin resistance, two CYP6 genes belonging to a cluster of P450s in chromosome 1 and two CYP9Js belonging to a large cluster of P450s on chromosome 3 were previously identified as affected by CNV associated with deltamethrin resistance (Faucon et al., 2015). Genes associated with bendiocarb resistance included fifteen P450s, three GSTs and two CCEs. P450s included all genes of the CYP6 cluster located on chromosome 1 and four genes of the large CYP9J cluster located on chromosome 3. The CYP6‐like *AAEL009018* located on chromosome 1 was specifically associated with bendiocarb resistance with a marked CNV increase in both F0 and F2 survivors. Genes associated with fenitrothion resistance included eleven P450s, three GSTs, three glycosyltransferases (UDPGTs), two CCEs and one sulfotransferase. Genes specifically associated with fenitrothion resistance included the sulfotransferase *AAEL004557*, the P450 *CYP304B3* on chromosome 1, five genes on chromosome 2 (*CYP6N17*, *CYP6Z8*, *CYP6M5*, *GSTX2*, UDPGT *AAEL000687*) and the UDPGT *AAEL005468* located at the end of chromosome 3.

### 3.4 Selection imprints and polymorphisms

Over 85% of sequenced reads were successfully mapped to AaegL5 genome allowing the detection of more than 40,000 polymorphisms. Among them, 24,714 passed quality filters and were polymorphic across conditions (Table S3). These polymorphisms mostly included substitutions (96.6%) located in targeted regions. The mean distance between two consecutive markers was ~50 kb.

**Figure 5:**
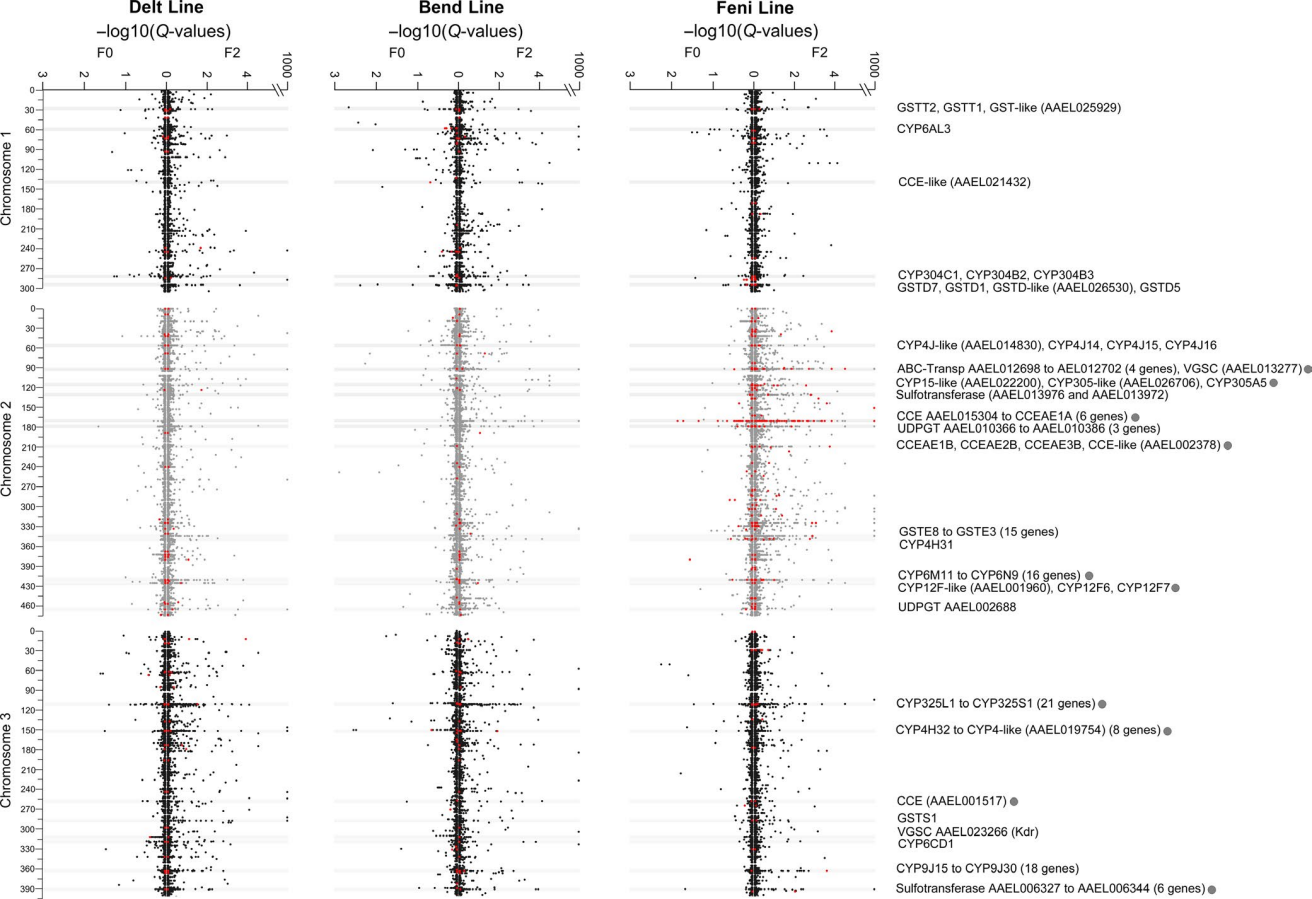
Genomic regions associated with insecticide resistance. For each line, regions associated with resistance were identified based on the presence of polymorphisms showing allele frequency variations from F0 to F2 survivors associated with resistance (red dots, see Section 2 for allele frequency filtering conditions) and their selection signature inferred from a Bayesian genome scan approach. For each line, Bayescan *Q* values were computed separately in F0 (left arm) and F2 conditions (right arm). *Q* values = 0 were fixed at 10^–1,000^ for better clarity. Horizontal grey lines indicate genomic regions showing both strong selection signatures and differential polymorphisms within 50 kb of candidate resistance genes. Grey dots indicate genomic regions carrying genes affected by differential nonsynonymous variations associated with resistance as shown in Figure 6

Filtering these polymorphisms based on their expected frequency variations from F0 to F2 in each line (see Section 2 for filtering conditions) allowed identifying 302 differential polymorphisms (1.23%) associated with insecticide resistance. Most of them were line‐specific with only three of them shared between the Feni and Bend lines. Combining this filtering approach with a *F*_ST_‐based Bayesian genome scan approach allowed detecting multiple genomic regions both carrying differential polymorphisms and showing low *Q* values in both F0 and F2 samples in each line (Figure 5 and Table S4). Most of them were located in proximity of genes potentially involved in metabolic resistance and included genes carrying nonsynonymous polymorphisms associated with resistance (see below). Among these regions, five were located on chromosome 1, including two GST clusters and one P450 clusters. The P450 cluster (three CYP304 genes at ~287 Mb) showed a pronounced selection signature for the Delt and Bend lines while the GST cluster located at ~300 Mb showed a strong selection signature in the Bend line. Several regions were also detected on chromosome 2. One ABC transporter cluster (four genes at ~90 Mb), one sulfotransferase cluster (two genes at 134.15 Mb) and two CCE clusters (six genes at ~174 Mb and 4 genes at ~214 Mb) showed strong selection signatures in the Feni line. The large GST cluster (15 GSTE genes at ~351.5 Mb) showed a selection signature in Feni and Delt lines while the large CYP6 cluster (16 genes at ~419.2 Mb) appeared less specific. Among regions identified in chromosome 3, the two large P450 clusters (21 CYP325 genes at ~111.6 Mb and 18 CYP9J genes at ~368.5 Mb) and the sulfotransferase cluster (six genes at 396.8 Mb) showed selection signatures all lines. Finally, despite the 81 polymorphisms detected in the voltage‐gated sodium channel gene (gene *AAEL023266 *at ~316 Mb in chromosome 3), only a moderate selection signature was detected at this locus in F0 individuals of the Delt line, probably due to the poor enrichment of Kdr mutations in F2 individuals surviving low insecticide dose.

**Figure 6:**
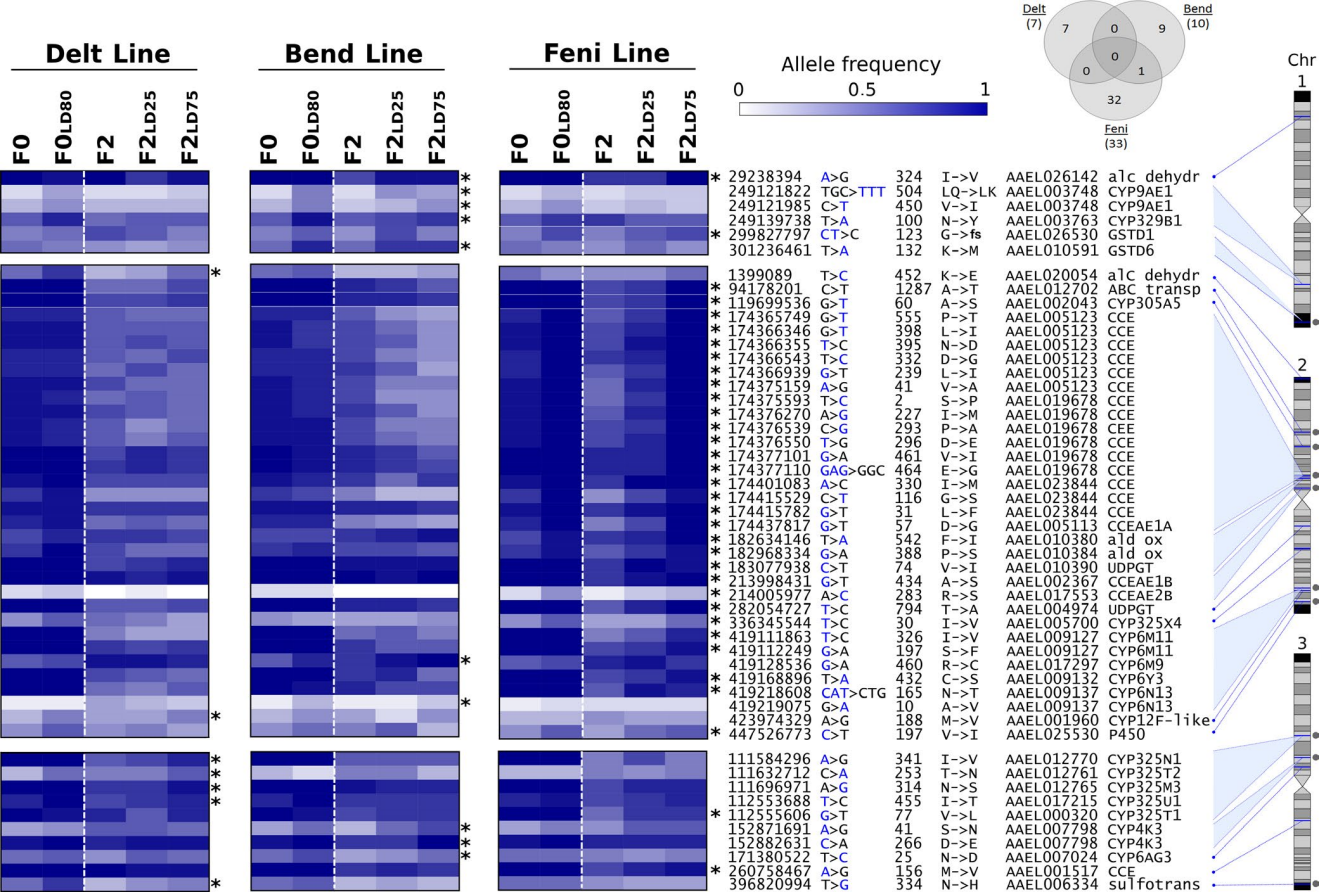
Nonsynonymous polymorphisms associated with insecticide resistance. The heat map shows the allele frequency variations of all nonsynonymous polymorphisms affecting detoxification genes associated with resistance from the frequency filtering approach. Stars indicate variations associated with resistance in each line. For each variation, the allele represented on the heat map is indicated in blue. Allele frequency data obtained from F0 Guy‐R are shown for each line for better clarity. Grey dots indicate nonsynonymous polymorphisms located in genomic regions showing selection signatures as shown in Figure 5. For each variation, the following annotations are shown: genomic location, reference nucleotide > variant nucleotide, amino acid position, amino acid change (fs, frameshift), gene accession number and gene name. The Venn diagram shows the number of variations identified in each line

Among differential polymorphisms associated with resistance, 50 were nonsynonymous and affected detoxification enzymes (Figure 6 and Table S4). Most of them were located in genomic regions showing selection signatures associated with resistance. All of them were line‐specific except the I324V mutation affecting the alcohol dehydrogenase gene *AAEL026142,* which was identified in the Bend and Feni lines. Seven nonsynonymous polymorphisms were associated with resistance in the Delt line. These affected the alcohol dehydrogenase *AAEL020054* and the P450 *AAEL001960* in chromosome 2 together with four clustered P450s and 1 sulfotransferase in chromosome 3. Ten nonsynonymous polymorphisms were associated with resistance in the Bend line affecting eight distinct genes. Four were located on chromosome 1: the alcohol dehydrogenase *AAEL026142*, the P450s *CYP9AE1* and *CYP329B1*, and *GSTD6*. Two were located on chromosome 2: *CYP6M9* and *CYP6N13*. Three were located on chromosome 3: the P450s *CYP4K3* (2 variations) and *CYP6AG3*. Finally, more than 30 nonsynonymous polymorphisms were associated with resistance in the Feni line affecting 21 distinct genes. On chromosome 1, this included the alcohol dehydrogenase *AAEL026142* and *GSTD1* for which a coding frameshift was negatively associated with resistance. Multiple isolated genes were affected on chromosome 2, including 1 ABC transporter, few P450s and 1 UDPGT. Two CCE clusters located within regions showing strong selection signatures (at ~174 and ~214 Mb) were also affected with the first one being affected by 17 nonsynonymous polymorphisms. Three P450s (*CYP6M11*, *CYP6Y3* and *CYP6N13*) located within a large CYP6 cluster (at ~419 Mb) were also affected on chromosome 2. Only two genes (the P450 *CYP325T1* and the CCE *AAEL001517*) were affected on chromosome 3.

## 4 DISCUSSION

Natural populations experience a variety of selective pressures often leading to the expression of complex adaptive phenotypes. In mosquitoes transmitting human diseases, an over‐reliance on chemical control has resulted in the rapid selection and spread of alleles conferring resistance to various insecticides, often leading to multi‐resistance phenotypes (Li et al., 2007; Moyes et al., 2017; Ranson et al., 2009). As opposed to target‐site mutations which are specific to a given insecticide mode of action, the complexity and redundancy of insect detoxification systems underlying metabolic resistance make it less predictable and can lead to the selection of various resistance alleles depending on the local context (Feyereisen, 2005; Li et al., 2007). Most insecticide resistance studies using field mosquito populations focus on resistance mechanisms to a given insecticide. However, such studies do not fully discriminate alleles associated with resistance to different insecticides, which may lead to false positives. In this context, the present study shows that the specificity of resistance alleles' detection can be improved by combining simple genetic crosses and targeted DNA pool sequencing, while maintaining experimental work and sequencing costs at reasonable levels.

### 4.1 Controlled crosses for enhancing the detection of resistant alleles

Bioassays confirmed the high resistance of the F0 Guy‐R composite population from French Guiana to deltamethrin and its moderate resistance to the two other insecticides. As expected, the introgression of susceptible alleles by controlled crosses strongly reduced deltamethrin resistance in F2 individuals. This suggests that resistance alleles approaching fixation in the initial F0 Guy‐R population were less present in F2 individuals, thus facilitating their subsequent phenotype association testing. This was confirmed by the strong decrease in kdr mutations frequencies observed from F0 to F2 individuals.

Cross‐resistance patterns of F2 lines supported the enrichment of specific resistant alleles in each line. This was supported by the higher kdr mutation frequencies observed in F2 survivors of the Delt line. However, cross‐resistance still occurred between lines suggesting that only two generations of recombination are likely not enough to break genetic associations between alleles conferring resistance to distinct insecticides or that particular alleles are conferring resistance to multiple insecticides. This was previously shown in *Anopheles* mosquitoes and *Drosophila melanogaster* where particular detoxification enzymes can metabolize multiple insecticides from different chemical families (Daborn et al., 2007; Edi et al., 2014; Mitchell et al., 2012; Riveron et al., 2014).

### 4.2 CNV affecting detoxification enzymes are associated with resistance

Metabolic resistance is frequently associated with the over‐expression of detoxification enzymes having the ability to degrade and/or sequester insecticides (Hemingway et al., 2004; Li et al., 2007). Although changes in gene expression can result from cis‐ or trans‐mediated transcriptional or post‐transcriptional regulation, CNV also impact gene expression. Initially restricted to organophosphate resistance in *Culex pipiens* (Raymond, Chevillon, Guillemaud, Lenormand, & Pasteur, 1998), recent genomic studies confirmed the key role of CNV in metabolic resistance to various insecticides in mosquitoes (Faucon et al., 2015, 2017; Lucas et al., 2019; Weetman, Djogbenou, & Lucas, 2018). Such key role is not surprising as the locus mutation rate is typically far higher for CNV than for mutation (Campbell & Eichler, 2013). In addition CNV events are promoted by the presence of transposable elements which account for a large part of most mosquito genomes (~50% in *Ae. aegypti* genome, Nene et al., 2007). Furthermore, CNV have a direct impact on gene expression level (i.e., gene dosage effect) without necessarily altering protein function (Gamazon & Stranger, 2015). This suggests that the initial selection of homologous detoxification gene duplications may only be counterbalanced by metabolic costs related to increased protein production. Finally, it has been shown in yeast that a specific environmental change can stimulate the occurrence of CNV affecting genes involved in adaptation to the novel environment (Hull, Cruz, Jack, & Houseley, 2017). Considering that the proposed transcription‐related mechanism depends on promoter activity and that detoxification enzymes are frequently inducible by xenobiotics, such mechanisms might also have contributed to the selection of CNV‐mediated metabolic resistance to insecticides in mosquito populations.

Our study identified 39 detoxification genes affected by CNV in association with resistance to insecticides. Although the F0 Guy‐R composite population from French Guiana exhibits a high resistance to the pyrethroid deltamethrin, only few CNV were found associated with resistance to this insecticide and most of them did not show a strong dose–response in F2 individuals. Such low CNV signal was likely caused by the presence of kdr mutations which are known to be of significant importance in deltamethrin resistance in *Ae. aegypti* (Haddi et al., 2017; Moyes et al., 2017; Smith et al., 2016). This was confirmed by the high kdr mutations frequencies found in F2 individuals surviving high dose of deltamethrin. However, our data also supported the added value of an increased gene copy number of detoxification enzymes in deltamethrin resistance. This was particularly apparent in F0 survivors of the Delt line for which kdr mutations approached fixation. Indeed, these survivors showed a strong CNV increase affecting two P450s clusters: a CYP6 cluster located on chromosome 1 and a CYP9J cluster on chromosome 3. The over‐expression of P450s from these two clusters was previously associated with pyrethroid resistance in *Ae. aegypti* (David et al., 2013; Moyes et al., 2017; Smith et al., 2016) and in *Aedes albopictus* (Ishak et al., 2016). Some of these genes, such as *CYP6BB2*, *CYP9J28* and *CYP9J32,* have been functionally validated as able to metabolize pyrethroid insecticides (Kasai et al., 2014; Stevenson, Pignatelli, Nikou, & Paine, 2012), and CNV affecting these P450s were observed in field populations resistant to deltamethrin (Faucon et al., 2015, 2017). Read coverage profiles suggest that a single genomic amplification of ~140 kb affects the CYP6 cluster while the genomic architecture of the amplification affecting the CYP9J cluster appears more complex. Sequencing these genomic regions in individual mosquitoes from various locations will allow deciphering their polymorphism in natural populations (Lucas et al., 2019). Finally, the identification of CNV affecting multiple GSTs in association with deltamethrin resistance supported their role in deltamethrin resistance (David et al., 2005; Kostaropoulos, Papadopoulos, Metaxakis, Boukouvala, & Papadopoulou‐Mourkidou, 2001; Lumjuan et al., 2011; Vontas, Small, & Hemingway, 2001).

As compared to deltamethrin, more detoxification genes were affected by CNV associated with bendiocarb and fenitrothion resistance (Bend and Feni lines). Actually, even though resistance levels to these two insecticides were lower in the initial Guy‐R population, the absence of mutations affecting the acetylcholinesterase gene (Ace1 gene) in *Ae. aegypti* because of genetic constraints (Weill et al., 2004) may have strengthened their association with the resistance phenotype in the Bend and Feni lines. Among genes affected by CNV associated with bendiocarb resistance, the CYP6 *AAEL009018* located on chromosome 1 showed a strong and specific association with bendiocarb. The over‐transcription of this gene was previously identified in multi‐resistant populations from the Caribbean (Bariami, Jones, Poupardin, Vontas, & Ranson, 2012) but also in Malaysian populations showing resistance to pyrethroids and carbamates (Ishak et al., 2017). The weak association of this gene with deltamethrin resistance observed in our study supports its role in carbamate resistance.

Several CNV affecting various detoxification genes were associated with resistance to fenitrothion. The genes *CYP6N17*, *CYP6Z8* and *CYP6M5*, *GSTX2* and the UDPGT *AAEL000687* were specifically associated with fenitrothion resistance. Noteworthy, orthologous genes were also found highly over‐transcribed in a Greek *Ae. albopictus* strain selected with the organophosphate temephos, supporting their potential contribution in organophosphate resistance (Grigoraki et al., 2015). The amplification of a CCE cluster known to play a key role in temephos resistance (Grigoraki et al., 2016) was not detected in our study, most likely because this CCE amplification is not present in French Guiana as suggested by previous studies (Faucon et al., 2015, 2017).

Overall, the present study supports the contribution of CNV in the over‐expression of detoxification enzymes conferring insecticide resistance in mosquitoes. The functional validation of these CNV markers through genome editing (e.g., CRISPR/Cas9) or gene knock‐down (e.g., RNAi) will allow identifying gene duplications most contributing to the resistance phenotype. Studying their frequency variations and their structural polymorphism in resistant populations from various continents will allow assessing their usefulness as novel DNA markers for tracking metabolic resistance worldwide. Such DNA marker will have the advantage of allowing the concomitant genotyping of target‐site mutations and metabolic resistance alleles from single mosquito specimens.

### 4.3 Selection signatures and nonsynonymous variations associated with resistance

Combining allele frequency filtering and *F*_ST_‐based selection signature detection allowed identifying multiple genomic regions associated with insecticide resistance in each line, supporting the multigenic nature of resistance. Some of them appeared specifically associated with resistance to a given insecticide in F2 lines, confirming the added value of controlled crosses for enhancing the specificity of resistance loci detection. Resistance‐associated loci were often located in close proximity to detoxification genes previously associated with insecticide biodegradation or found over‐expressed in resistant populations (reviews in Moyes et al., 2017; Smith et al., 2016), supporting the robustness of our dual filtering approach. However, a few regions showing strong selection signatures were identified near genes rarely associated with resistance in *Ae. aegypti*. This included multiple P450s from the CYP325, CYP4 and CYP12 families but also GSTs, UDPGTs, ABC‐transporters and sulfotransferases, which may all be involved in insecticide metabolism pathways. Most of these regions included detoxification genes carrying nonsynonymous variations associated with resistance suggesting that these resistant loci may reflect the selection of particular detoxification enzyme variants. Even though most resistance studies focused on the identification of over‐expressed detoxification genes, the selection of particular variants leading to an increased insecticide metabolism rate can also contribute to the overall resistance phenotype as demonstrated in the malaria vector *An. funestus* (Ibrahim et al., 2015; Riveron et al., 2014). The deletion leading to a frameshift coding in *GSTD1* is also of particular interest as the functional allele was specifically associated with resistance to fenitrothion. This enzyme has been shown to catalyse DDT dechloration and to be expressed in detoxification tissues in *An. gambiae* (Ingham et al., 2014; Ranson et al., 1997), supporting its role in insecticide resistance. Also of interest are the multiple nonsynonymous variations associated with fenitrothion resistance affecting a cluster of CCE genes located at 174 Mb in chromosome 2. Among them, the gene *CCEae3A* (*AAEL023844*) has been shown to sequester and metabolize the organophosphate temephos in both *Ae. aegypti* and *Ae. albopictus* (Grigoraki et al., 2016), and the over‐expression of this CCE gene through increased gene copy number was associated with temephos resistance (Faucon et al., 2015; Poupardin, Srisukontarat, Yunta, & Ranson, 2014). However, no CNV were detected for this gene in the present study, suggesting that the selection of CCEae3A variants may also contribute to organophosphate resistance. This hypothesis is also supported by the previous identification of point mutations in *CCEae3A* for which docking simulations predicted an impact on temephos binding (Poupardin et al., 2014). Although none of these mutations were associated with resistance in our data set, other nonsynonymous mutations associated with resistance identified were located near the catalytic triad (e.g., I330M in *CCEae3A* and D332G in *AEL005123*) or the active site (e.g., P293A in *AAEL019678*). Although further work is required to validate the functional role of nonsynonymous variations of detoxification enzymes, the present study provides a comprehensive data set for better understanding the contribution of detoxification enzyme variants in insecticide resistance.

## 5 CONCLUSIONS

Although insecticide resistance is often presented as a monogenic adaptation in response to a strong selection pressure, it frequently results from the accumulation of multiple physiological and metabolic changes often leading to complex phenotypes. Because of their nature, target‐site mutations are usually well characterized in mosquitoes and can typically be genotyped by simple PCR‐based molecular assays (Moyes et al., 2017; Smith et al., 2016). In contrast, genomic changes associated with metabolic resistance are far more difficult to characterize for various reasons: First, metabolic resistance alleles frequently co‐occur with target‐site mutations, thus weakening their association with the overall resistance phenotype. Second, the complexity and redundancy of insect detoxification pathways may lead to the selection of multiple and diverse alleles through local adaptation. Third, increased insecticide metabolism can be the consequence of multiple and additive genetic changes including the over‐expression of detoxification enzymes through up‐regulation or increased gene copy number but also nonsynonymous polymorphisms causing structural changes of these enzymes.

Although massive parallel sequencing is a powerful tool for untangling the complexity of the genetic bases of metabolic resistance, its association with a well‐thought experimental design is required to reduce both false negatives and false positives. Here, we demonstrated that combining simple genetic crosses with pool targeted DNA‐seq can enhance the specificity of resistance alleles' detection and produce high coverage sequence data while maintaining experimental work and sequencing costs at an acceptable level (~650€/condition including wet‐lab costs vs. ~5,000€/condition for a standard whole genome sequencing approach based on 30 individuals sequenced at ~2× coverage per condition and a 1.3 Gb genome size). Our results also suggest that eliminating the effect of target‐site mutations by controlled crosses or gene editing should improve the power of genotype–phenotype association studies targeting metabolic resistance alleles. Considering the global threat of insecticide resistance on vector control and the decades that will be necessary for the full deployment of insecticide‐free strategies, identifying a set of DNA resistance markers reflecting the variety of resistance mechanisms occurring in natura still represents a key step for improving the tracking and management of insecticide resistance in mosquitoes.

## CONFLICT OF INTEREST

None declared.

## ETHICAL APPROVAL

Blood feeding of adult mosquitoes was performed on mice. Mice were maintained in the animal house of the federative structure Environmental and Systems Biology (BEeSy) of Grenoble‐Alpes University agreed by the French Ministry of Animal Welfare (agreement no. B 38 421 10 001) and used in accordance to European Union laws (directive 2010/63/UE). The use of animals for this study was approved by the ethic committee ComEth Grenoble‐C2EA‐12 mandated by the French Ministry of higher Education and Research (MENESR).

## Data Availability

The sequence data from this study have been deposited to the European Nucleotide Archive (ENA; http://www.ebi.ac.uk/ena) under the accession number PRJEB30945 (Cattel et al., 2019).
