# Supplementary figures and images for: Combining genetic crosses and pool targeted DNA‐seq for untangling genomic variations associated with resistance to multiple insecticides in the mosquito Aedes aegypti
Source: Evol Appl. 2019 Sep 27;13(2):303–17. doi: 10.1111/eva.12867 (PMC6976963; doi:10.1111/eva.12867)

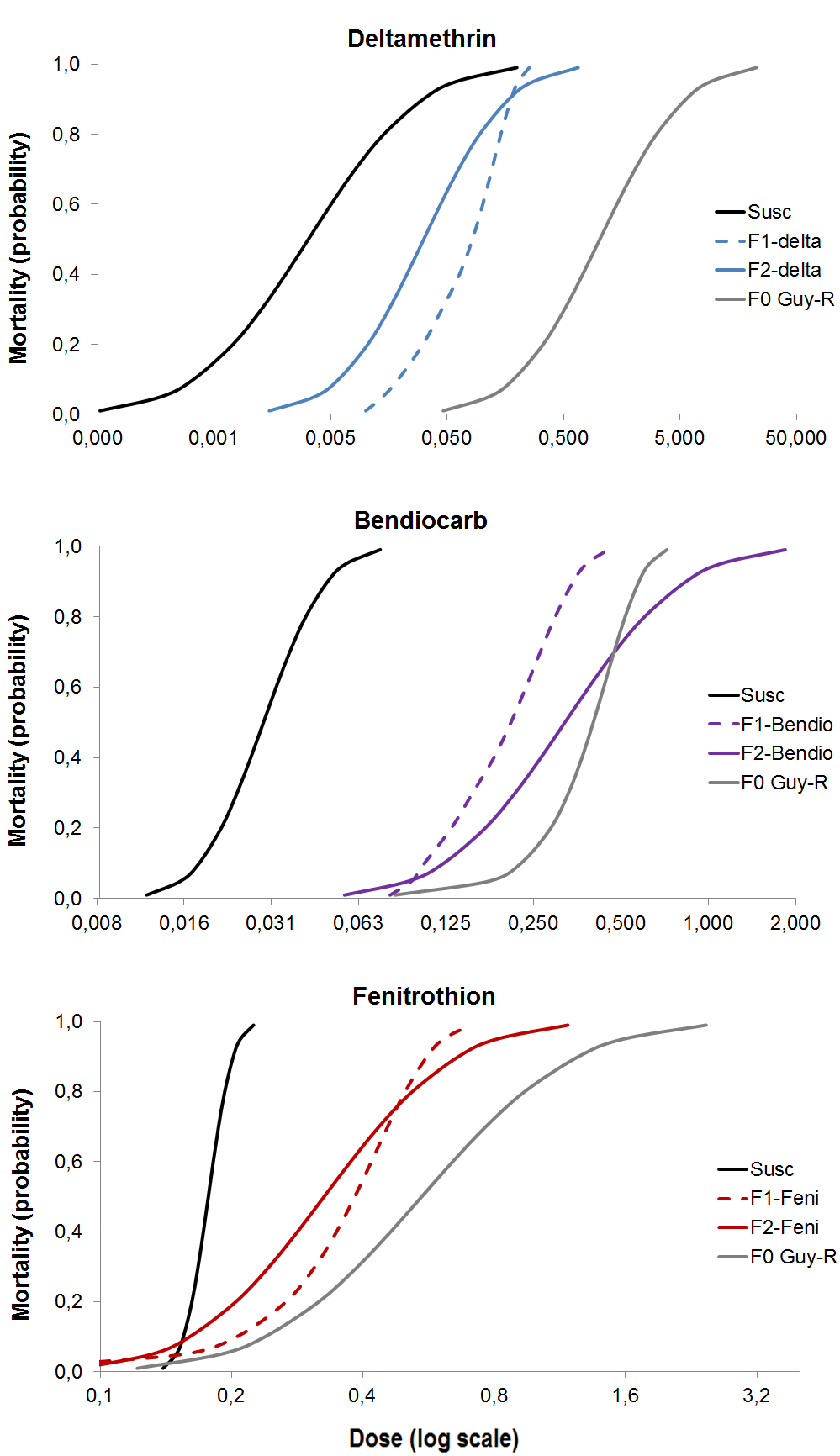

Supplement: Supplementary file 1 [file EVA-13-303-s001.tif]

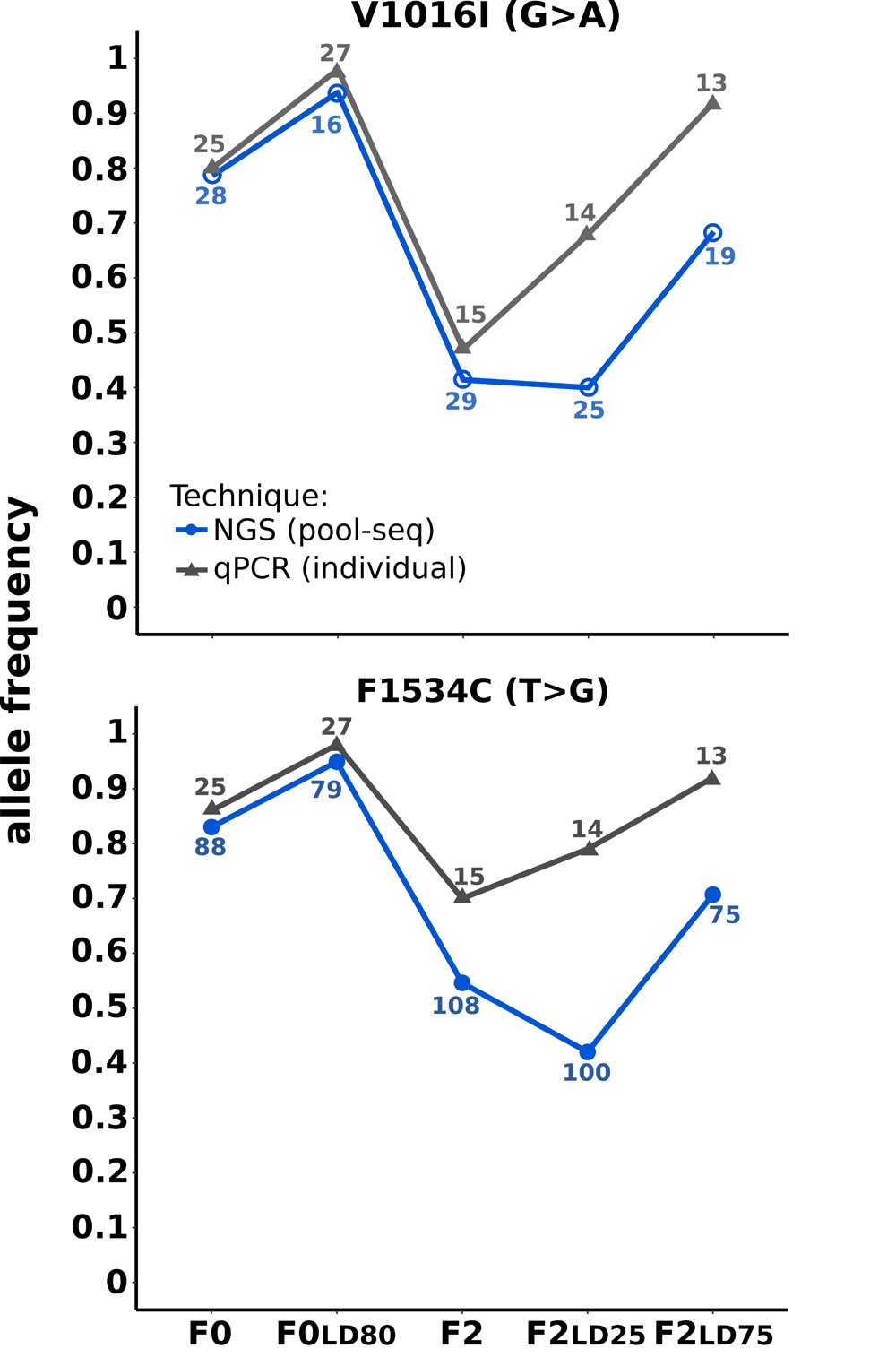

Supplement: Supplementary file 2 [file EVA-13-303-s002.tif]
